# Lazarus Effect of High Dose Corticosteroids in a Patient With West Nile Virus Encephalitis: A Coincidence or a Clue?

**DOI:** 10.3389/fmed.2019.00081

**Published:** 2019-04-25

**Authors:** A. Arturo Leis, David J. Sinclair

**Affiliations:** ^1^Center for Neuroscience and Neurological Recovery, Methodist Rehabilitation Center, Jackson, MS, United States; ^2^Mississippi Baptist Medical Center, Jackson, MS, United States

**Keywords:** West Nile virus encephalitis, neuroinvasive disease, autoimmunity, post-infectious, neuroinflammation

## Abstract

West Nile virus (WNV) causes severe neuroinvasive disease in humans characterized by meningitis, encephalitis, and acute flaccid paralysis (poliomyelitis variant). In neuroinvasive disease, WNV infection of neurons resulting in neuronal loss is generally presumed to be the anatomical substrate for the high morbidity and mortality. However, on a molecular level, WNV infection also results in a significant upregulation of important proinflammatory molecules that have been reported to promote neuroinflammation and cytotoxicity. Currently, there is no specific treatment for the neurological complications of WNV infection. We present a 71-year-old woman who developed WNV infection that rapidly progressed to severe generalized weakness and encephalitis manifesting with bulbar signs (dysphagia, dysarthria) and persistent delirium and stupor. Consciousness remained impaired for 9 days and then she received a 5-day course of high-dose intravenous methylprednisolone (1,000 mg daily). After the first day, voluntary movement and spontaneous eye-opening increased and by the end of the second day, she was awake and responding to commands. Thereafter, she remained awake and responsive. Although the rapid improvement from stupor to wakefulness following treatment with an anti-inflammatory immunosuppressant could merely be coincidence, since these observations are of one patient, it may also provide a clue that in some cases of WNV neuroinvasive disease a *post-infectious pro-inflammatory state*, rather than neuronal loss, may also contribute to morbidity. Further clinical trials are warranted to determine if high dose corticosteroids and other drugs that can alter this neuro-inflammatory cascade may be potentially beneficial in the treatment of WNV neuroinvasive disease.

## Introduction

West Nile virus (WNV) is a neurotropic virus that causes a spectrum of human disease ranging from a febrile illness (WNV fever), often described as a “summer flu,” to severe neuroinvasive disease classified as meningitis, encephalitis, or acute flaccid paralysis. In neuroinvasive disease, neuronal loss attributed to direct viral invasion of neurons is the presumed anatomical substrate for the high morbidity and mortality. However, on a molecular level, WNV infection in humans induces a significant upregulation of important proinflammatory proteins, including glial signaling protein S100B (1, 2), intracellular protein high-mobility group box-1 (HMGB1) (3) and osteopontin (OPN) (4), a multifunctional cytokine. These proinflammatory molecules are increased in human serum or cerebrospinal fluid (CSF) following the acute infection and have been reported to promote neuroinflammation and to contribute to cytotoxicity in the central nervous system (CNS). There is no specific treatment or vaccine currently approved in humans, and the standard remains supportive care.

We present the case of an elderly woman who developed WNV encephalitis and prolonged delirium (confusion with reduced awareness of surroundings) and stupor (requiring repeated stimulation to be aroused) who showed marked improvement in consciousness within 2 days of high-dose intravenous methylprednisolone. The rapid improvement from stupor to wakefulness following treatment with an immunosuppressant allows us to hypothesize that a pathogenic *post-infectious pro-inflammatory state*, rather than neuronal loss, may contribute to morbidity in some cases of WNV neuroinvasive disease.

This retrospective case report was carried out in accordance with the Institutional Review Boards for Human Research at Methodist Rehabilitation Center and the Baptist Hospital, Jackson, MS. The subject's spouse and family gave written informed consent in accordance with the Declaration of Helsinki. The protocol was approved by the respective Institutional Review Boards. Written informed consent was also obtained from the patient for the publication of this case report.

## Case Report

A 71-year-old female obstetrician with no medical history, except hysterectomy and knee surgery, presented to the emergency department (ED) after 1½ days of worsening fatigue, fever, chills, headache, generalized weakness, difficulty walking, and maculopapular rash in both legs. Her neurological status declined while in the ED and she was treated with broad spectrum antibiotics and acyclovir and admitted to the progressive care unit for close monitoring. Initial neurologic examination revealed an elderly febrile woman with a temperature of 102.7°F and nuchal rigidity who was confused, disoriented, following commands poorly, and non-verbal, with eyes open but “glazed.” She had prominent generalized weakness (Medical Research Council 2/5 in proximal muscles and 3/5 in distal muscles) with decreased spontaneous movement in all limbs. The neurologist concluded: “Patient is critically ill with fulminant neurological deterioration with potential for further deterioration and possibly death.” A CT scan and MRI of the brain were normal and spinal tap performed the day of admission showed CSF pleocytosis with white cell count of 720 mm (neutrophils 88%, lymphocytes 7%), protein 174 g/dL, and glucose 65 mg/dL. Meningoencephalitis and arbovirus panels were ordered, including serum and CSF WNV antibody tests (ELISA). The patient's neurological status continued to decline and she developed severe dysphagia and became stuporous, requiring stimulation to remain awake. Given her worsening mental status and inability to clear secretions she was transiently intubated to protect her airways and transferred to the neurosciences intensive care unit. After nasogastric tube insertion, she was extubated because of the low risk of aspiration and the constant supervision from family members. The WNV IgM antibody tests were reported positive on about hospital day 5 (WNV CSF IgM 10.87, normal ≤ 0.89 IV; WNV CSF IgG 0.10, normal ≤ 1.29 IV; WNV serum IgM 4.52, normal < 0.80 ratio). IgM and IgG results for St. Louis encephalitis, California encephalitis, Eastern equine, and Western equine viruses were negative. For a total of 9 days she remained largely obtunded with a depressed level of consciousness, often requiring non-invasive tactile stimulation (e.g., monofilament tickle to inside of nostril) for temporary arousal. Cranial nerve examination revealed dysconjugate gaze, left ptosis, decreased gag reflex, poor tongue protrusion, bilateral hand tremor, involuntary myoclonic-like jerks in right shoulder muscles (mainly trapezius), and equivocal Babinski signs. An EEG showed diffuse 4-7 Hz theta activity consistent with diffuse cerebral dysfunction, but no epileptiform activity. With prognosis still uncertain, a trial of high dose intravenous methylprednisolone (1 gram every morning × 5 days) was initiated. By the end of day 1 infusion, the patient's voluntary movements and spontaneous eye-openings increased; by the end of day 2 infusion, the patient was awake and responding to commands. Improved mentation persisted and following completion of the steroid infusions, she was transferred to a regular care bed. A serum cytokine panel (interleukin (IL)-2, IL-2 receptor CD25 soluble, IL-12, interferon-gamma, IL-4, IL-5, IL-10, IL-13, IL-17, IL-1 beta, IL-6, IL-8, and tumor necrosis factor alpha) drawn the day after completing the 5-day course of methylprednisolone was normal except for a borderline S100B protein (90 ng/L, normal 0–96).

## Discussion

We present an unusual case of a Lazarus-like effect of high dose corticosteroids in a stuporous woman with WNV encephalitis. There are two questions that need to be answered regarding the rapid transformation from stupor to wakefulness; is there sufficient evidence for a causal relationship with the pharmacologic intervention, and, if so, what is the therapeutic mechanism? Although the disease course of WNV neuroinvasive disease may be variable, with some patients recovering and others progressing to coma or death, the outcomes of patients with neurological manifestations is generally poor. In one large series of 57 patients with neurological sequelae of WNV encephalitis, 10 patients died (case-fatality rate 18%), 37 had persistent neurological deficits, the mean length of stay in an ICU was 28 days, only 9 recovered fully, and only 13 (23%) were discharged home without extra support (5). In another study that collected longitudinal information about the recovery process in 55 subjects with WNV neuroinvasive disease, factors that predicted severity or long-term recovery of neurological function included age (older individuals had more neurologic dysfunction at follow-up examinations), gender (females had a poorer prognosis for recovery from coma), and neurological deficits at presentation (those with depressed level of consciousness and cranial nerve deficits predicted poorer recovery, particularly with regard to cognition) (6). Our patient had multiple risk factors for poorer recovery: she was elderly, encephalopathic, and had cranial nerve deficits (dysconjugate gaze, severe dysphagia, dysarthria). Hence, it seems reasonable to postulate that administration of high dose corticosteroids may have played a causal rather than a coincidental role in the rapid transformation from stupor to wakefulness. This conclusion is further supported by published reports of improvement in patients with WNV neuroinvasive disease after the use of steroids. In one case series of 14 patients with acute WNV meningoencephalitis, the authors suggested that intravenous dexamethasone may have been the reason for shortening of the acute phase of WNV disease and hastening patient recovery (7). High-dose steroids were also used to successfully treat a patient with WNV-associated acute flaccid paralysis (8). In addition, 2 patients with acute flaccid paralysis and brainstem involvement, including progressive seventh nerve palsies, showed clear improvement in brainstem symptoms and facial paralysis within 24 h of treatment with high-dose intravenous steroids (9).

Although the use of corticosteroids in patients with WNV neuroinvasive disease seems counterintuitive, with concern that immunosuppressive effects may promote viremia and worsen outcome, there is compelling evidence that WNV is rapidly cleared by effective innate and adaptive immune responses within several days of the onset of viremia. Indeed, the first isolation of infectious WNV from a human case-patient in the United States occurred in late 2002, four summers after WNV gained entry into North America and after a total of 2,671 cases of human illness had been reported to the Centers for Disease Control and Prevention (CDC) (10). This patient was immunocompromised and was undergoing chemotherapy for a non-Hodgkin B-cell lymphoma. Hence, the first instance of WNV recovered from a person in the United States was facilitated by the patient's immunological suppression. To date, it is commonly accepted that infectious WNV cannot be isolated from humans with a normal immune system following the production of neutralizing antibodies. Additional support that immunosuppressive therapy does not worsen outcome comes from large scale trials in dengue, a related Flavivirus. A comprehensive review (2018) that included 13 studies enrolling 1,293 participants on the effectiveness of corticosteroids in the treatment of dengue, found no evidence that administration of high doses of oral or intravenous corticosteroids resulted in viremia or significant adverse side effects (11). Indeed, therapeutic benefit was seen in multiple studies that used intravenous delivery of high doses and multiple doses of steroids. In contrast, clinical studies without administration of repeated high doses of steroids showed limited or no beneficial effect (11). Accordingly, a reasonable argument exists regarding the use of high dose steroids in the treatment of WNV neuroinvasive disease, since administration of such agents is unlikely to worsen outcome and may result in clinical improvement in some patients. However, to understand the therapeutic mechanism of the rapid conversion from stupor to wakefulness in this case, we need to be aware of the immunogenic changes that occur in the human body after WNV infection. There is abundant immunologic evidence that WNV infection causes a significant upregulation of several important proinflammatory molecules, including glial protein S100B (1, 2), high-mobility group box-1 (HMGB1) (3, 12), and osteopontin (OPN) (4). S100B, HMGB1, and OPN levels are increased in human serum or CSF post-infection, and correlate with severity of the WNV infection, being significantly higher in neuroinvasive disease than fever cases, and significantly higher than controls (1, 3, 4). S100B and HMGB1 also bind to the receptor for advanced glycation end products (RAGE), which serves as a major ligand for various immunoregulatory molecules. In the CNS, RAGE has been observed in neurons, microglia, and astrocytes, and multiple studies indicate a central and crucial role played by RAGE in neuroinflammation (12, 13). RAGE and OPN signaling initiates and maintains an inflammatory cascade that increases production of pro-inflammatory cytokines and chemokines. Indeed, multiple pro-inflammatory cytokines and interleukins have been found to be acutely and chronically increased in WNV survivors. In one study, 44 of 140 WNV patients (31%) that reported prolonged (> 6 months) post-infectious symptoms had significantly elevated pro-inflammatory proteins, including IL-2, IL-6, IL-12p70, granulocyte macrophage colony stimulating factor, interferon gamma, and interferon-gamma inducing protein 10 (14). Post-infectious CNS symptoms included impaired memory, difficulty concentrating, depression, anxiety, sleep disruption, and recurrent headaches. Patients also suffered from post-exertion fatigue, dizziness, altered sensation, arthralgias, and symptoms of dysautonomia (14). In another study, IL-17 in sera was shown to be elevated in WNV patients with acute neuroinvasive disease and also months or years later in those that had recovered from neuroinvasive disease (15). Hence, the post-infectious neuroinflammatory response may persist long after eradication of the virus and may have a clinical accompaniment in some WNV survivors. Importantly, treatment of infected neuronal cells with antibodies blocking pro-inflammatory mediators results in a significant reduction of WNV-mediated neuronal death (16), suggesting that such mediators may play a major role in the pathogenesis of WNV infection in the CNS (17).

## Conclusion

WNV is a neurotropic virus that can trigger an exaggerated immune response that promotes and amplifies neuroinflammation, which is considered a hallmark of WNV pathogenesis. This case supports the concept that neurological deficits may progress following the production of neutralizing antibodies, at a time when the virus has been cleared by effective innate and adaptive immune responses. In such cases, progression of neurological deficits is more likely to reflect injury from the downstream cascade of excitotoxic events and the secondary wave of neuroinflammation rather than neuronal loss *per se*. Greater awareness is needed that this post-infectious proinflammatory state may be potentially treatable. In our case, the rapid improvement following anti-inflammatory immune suppression may have been a coincidence, but we hope that this and other similarly evidenced cases will inspire others to initiate appropriate clinical trials to determine if high dose corticosteroids and other immunosuppressive drugs may be beneficial in the treatment of WNV neuroinvasive disease.

## Ethics Statement

This retrospective case report was carried out in accordance with the Institutional Review Boards for Human Research at Methodist Rehabilitation Center and the Baptist Hospital, Jackson, MS. The subject's spouse and family gave written informed consent in accordance with the Declaration of Helsinki. The protocol was approved by the respective Institutional Review Boards. Written informed consent was also obtained from the patient for the publication of this case report.

## Author Contributions

All authors listed have made a substantial, direct and intellectual contribution to the work, and approved it for publication.

### Conflict of Interest Statement

The authors declare that the research was conducted in the absence of any commercial or financial relationships that could be construed as a potential conflict of interest.

## Abbreviations

WNV: West Nile virus
OPN: osteopontin
IL: interleukin
HMGB1: high-mobility group box-1
RAGE: receptor for advanced glycation end products
CDC: Centers for Disease Control and Prevention
CSF: cerebrospinal fluid
CNS: central nervous system
ED: emergency department
IV: Interpretive value.

## References

[1] Leis AA Stokic DS Petzold A. Glial S100B is elevated in serum across the spectrum of West Nile virus infection. Muscle Nerve. (2012) 45:826–30. 10.1002/mus.23241 22581535

[2] Kuwar RB Stokic DS Leis AA Baif F Paulf AM Fratkind JD. Does astroglial protein S100B contribute to West Nile neuro-invasive syndrome? J Neurol Sci. (2015) 358:243–52. 10.1016/j.jns.2015.09.003 26382833

[3] Fraisier C Papa A Almeras L. High-mobility group box-1, promising serological biomarker for the distinction of human WNV disease severity. Virus Res. (2015) 195:9–12. 10.1016/j.virusres.2014.08.017 25197038

[4] Paul AM Acharya D Duty L Thompson EA Le L Stokic DS. Osteopontin facilitates West Nile virus neuroinvasion via neutrophil “Trojan horse” transport. Sci Rep. (2017) 7:4722. 10.1038/s41598-017-04839-7 28680095 PMC5498593

[5] Pepperell C Rau N Krajden S Kern R Humar A Mederski B. West Nile virus infection in 2002: morbidity and mortality among patients admitted to hospital in southcentral Ontario. CMAJ. (2003) 168:1399–405. 12771068 PMC155955

[6] Hart J Jr Tillman G Kraut MA Chiang HS Strain JF Li Y. West Nile virus neuroinvasive disease: neurological manifestations and prospective longitudinal outcomes. BMC Infect Dis. (2014) 14:248. 10.1186/1471-2334-14-248 24884681 PMC4020876

[7] Narayanaswami P Edwards L Hyde C Page C Hastings NE West Nile meningitis/ encephalitis: experience with corticosteroid therapy. Neurology. (2004) 62:A404.

[8] Pyrgos V Younus F. High- dose steroids in the management of acute flaccid paralysis due to West Nile virus infection. Scand J Infect Dis. (2004) 36:509–12. 10.1080/00365540410020659 15307586

[9] Leis AA Stokic DS. Neuromuscular manifestations of West Nile virus infection. Review. Front Neurol. (2012) 3:37. 10.3389/fneur.2012.00037 22461779 PMC3309965

[10] Huang C Slater B Rudd R Parchuri N Hull R Dupuis M Hindenburg A. first isolation of West Nile virus from a patient with encephalitis in the United States. Emerg Infect Dis. (2002) 8:1367–71. 10.3201/eid0812.020532 12498649 PMC2738499

[11] Rathnasiri Bandar SM Herath HMMTB Effectiveness of corticosteroid in the treatment of dengue - A systemic review. Heliyon. (2018) 4:e00816 10.1016/j.heliyon.2018.e00816 30258999 PMC6151849

[12] Wang H Ward MF Fan XG Sama AE Li W. Potential role of high mobility group box 1 in viral infectious diseases. Viral Immunol. (2006) 19:3–9. 10.1089/vim.2006.19.3 16553546 PMC1782047

[13] Juraneka J Raya R Banach M Rai V Receptor for advanced glycation end-products in neurodegenerative diseases. Rev Neurosci. (2015) 26:691–8. 10.1515/revneuro-2015-0003 26226128

[14] Garcia MN Hause AM Walker CM Orange JS Hasbun R Murray KO. Evaluation of prolonged fatigue post-West Nile virus infection and association of fatigue with elevated antiviral and proinflammatory cytokines. Viral Immunol. (2014) 27:327–33. 10.1089/vim.2014.0035 25062274 PMC4150370

[15] Acharya D Wang P Paul A Dai J Gate D Lowery JE. Interleukin-17A Promotes CD8^+^ T Cell cytotoxicity to facilitate West Nile virus clearance. J Virology. (2016) 91:e01529–16. 10.1128/JVI.01529-16 27795421 PMC5165211

[16] Kumar M Verma S Nerurkar VR Pro-inflammatory cytokines derived from West Nile virus (WNV)-infected SK-NSH cells mediate neuroinflammatory markers and neuronal death. J Neuroinflammation. (2010) 7:73 10.1186/1742-2094-7-73 21034511 PMC2984415

[17] Rossini G Landini MP Gelsomino F Sambri V Varani S. Innate host responses to West Nile virus: implications for central nervous system immunopathology. World J Virol. (2013) 2:49–56. 10.5501/wjv.v2.i2.49 24175229 PMC3785052

